# Farewell Prof. Hans‐Jürgen Rehm

**DOI:** 10.1111/1751-7915.12703

**Published:** 2017-02-27

**Authors:** Hermann J. Heipieper

**Affiliations:** ^1^ Department Environmental BiotechnologyHelmholtz Centre for Environmental Research –UFZLeipzigGermany

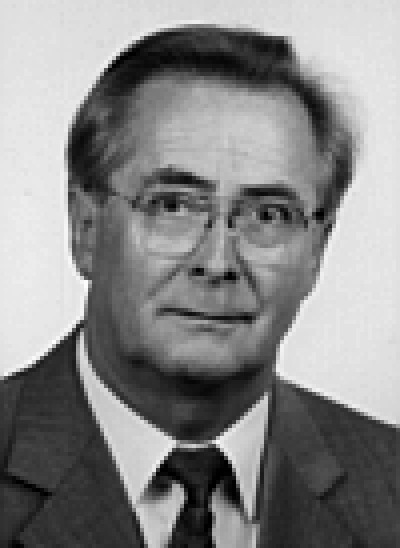

It is a great honor and duty for me to remember Prof. Dr. Hans‐Jürgen Rehm, born on 3 December 1927, who passed away in Münster, Germany, on 4 February 2017. Since 1970, he was director of the Institute of Microbiology at the University of Münster, Germany, where I had the great honor to be his Diploma student and also did my PhD under his supervision.

With his textbook ‘Industrielle Mikrobiologie’, first published in German in 1967 which was then translated in many language, he was of the main initiators of modern biotechnology in Europe.

Hans‐Jürgen Rehm was one of the founding members of the European Federation of Biotechnology and initiator of the European Conference on Biotechnology series.

In addition, together with Gerald Reed, Hans‐Jürgen Rehm was first editor of the famous series ‘Biotechnology – A Comprehensive Treatise’ in the late 1980s which is meanwhile a standard textbook series of biotechnology.

In Germany, he was head of the section ‘Biotechnology’ of the DECHEMA and made it an important international organization for industrial applications of biotechnological research.

Hans‐Jürgen Rehm was founding editor‐in‐chief of the journal Applied Microbiology and Biotechnology (AMB). With great gratitude we look back on a fulfilled life dedicated to academic teaching and science in the overall frame of European and worldwide biotechnology.

